# A Kinase Chaperones Hepatitis B Virus Capsid Assembly and Captures Capsid Dynamics *in vitro*

**DOI:** 10.1371/journal.ppat.1002388

**Published:** 2011-11-17

**Authors:** Chao Chen, Joseph Che-Yen Wang, Adam Zlotnick

**Affiliations:** Department of Molecular and Cellular Biochemistry, Indiana University, Bloomington, Indiana, United States of America; Institut Pasteur, France

## Abstract

The C-terminal domain (CTD) of Hepatitis B virus (HBV) core protein is involved in regulating multiple stages of the HBV lifecycle. CTD phosphorylation correlates with pregenomic-RNA encapsidation during capsid assembly, reverse transcription, and viral transport, although the mechanisms remain unknown. *In vitro*, purified HBV core protein (Cp183) binds any RNA and assembles aggressively, independent of phosphorylation, to form empty and RNA-filled capsids. We hypothesize that there must be a chaperone that binds the CTD to prevent self-assembly and nonspecific RNA packaging. Here, we show that HBV capsid assembly is stalled by the Serine Arginine protein kinase (SRPK) binding to the CTD, and reactivated by subsequent phosphorylation. Using the SRPK to probe capsids, solution and structural studies showed that SRPK bound to capsid, though the CTD is sequestered on the capsid interior. This result indicates transient CTD externalization and suggests that capsid dynamics could be crucial for directing HBV intracellular trafficking. Our studies illustrate the stochastic nature of virus capsids and demonstrate the appropriation of a host protein by a virus for a non-canonical function.

## Author Summary

A virus particle is a molecular machine that has evolved to self-assemble within the confines of a living cell. For hepatitis B virus (HBV), outside of a cell, the self-assembly process is very aggressive and consequently not specific for viral RNA. Here we show that HBV takes advantage of a host protein, SRPK, which acts like a molecular chaperone, to prevent the HBV core protein from binding RNA and to prevent the core protein from assembling at the wrong time and place. At the right time, SRPK can be removed in a regulated reaction to allow assembly. Once a virus is assembled, it must traffic to the right intracellular locale. Using SRPK, we show that HBV cores can transiently expose a segment of protein, normally inside the virus, that carries a signal for transport to the host nucleus. This is the first example we know of where a virus repurposes an enzyme for an alternative function. This sort of interplay between virus and host, where the virus hijacks and repurposes host proteins, is likely to be a common feature of viral infection.

## Introduction

Hepatitis B virus (HBV) is an enveloped DNA virus that causes liver damage and can lead to cirrhosis and liver cancer [1]. It has infected 2 billion people worldwide including 350 million chronic carriers [2], making it a major health concern, and also leading to social problems due to discrimination against the virus carriers where the disease is endemic.

Despite the extensive impact of HBV, there has been no effective treatment to eliminate the virus from carriers [3]. In part, this is because the virus life cycle is not fully understood. The viral infection starts with cell entry to release a viral core into the cytoplasm [4]. The core is a *T* = 4 icosahedral capsid of ∼35 nm diameter [5] containing a relaxed circular DNA (rcDNA) that is partially double-stranded and covalently bonded to a reverse transcriptase (RT). The core is transported to the nucleus where it releases the rcDNA, which is deproteinated [6] and ‘repaired’ by the host machinery to make a covalently-closed circular DNA (cccDNA) [7], [8]. Transcription of nuclear cccDNA generates the replication intermediate (pregenomic RNA, pgRNA) and other mRNAs [9]. PgRNA codes for core protein and RT. In the cytoplasm, core proteins encapsidate a pgRNA•RT complex to form immature HBV cores [10]. Maturation occurs when pgRNA is reverse-transcribed into rcDNA. Only mature cores are transported to the ER to acquire an envelope for subsequent secretion, or are delivered back to the nucleus for maintaining viral infection [11], [12].

**Figure 1:**
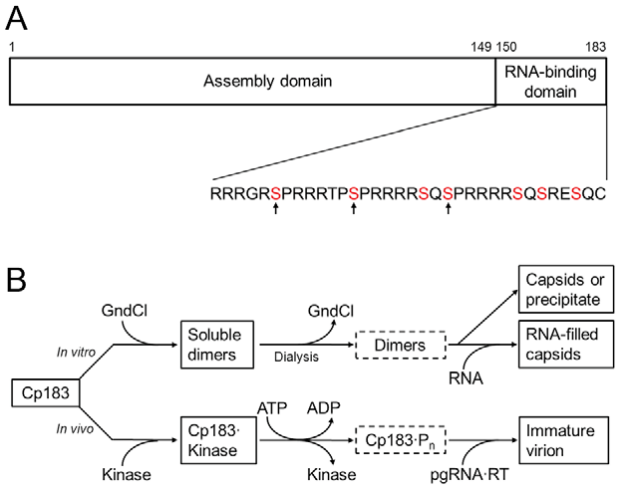
HBV core assembly and Cp183 phosphorylation. (A) A representation of the core protein sequence, showing the assembly domain (amino acids 1 to 149) and the nucleic acid-binding domain (amino acids 150 to 183). The primary sequence of the nucleic acid-binding domain contains 7 serines which are potential phosphorylation sites by SRPK (red). *In vivo*, phosphorylation of S155, S162, and S170 (indicated by arrows) confers specificity for pgRNA packaging [17], [18]. This panel is reproduced from reference [26] (Copyright © American Society for Microbiology, J. Virol., 2010, 84, 7174-84, doi:10.1128/JVI.00586-10). (B) Comparison between the *in vitro* and in vivo assembly pathways. Neither unphosphorylated nor phosphorylated Cp183 dimers (in dotted frames) are very soluble. Note the similar positions of GuHCl and kinase on the pathways. The kinase may also contribute to keeping Cp183 dimers soluble.

The viral core protein is a critical regulatory factor of the HBV life cycle. It is 183 amino acids in length, hence referred to as Cp183. The first 149 amino acids comprise the assembly domain [13] (Figure 1a). A core protein mutant consisting of this domain only (Cp149) can self-assemble *in vitro* to give particles whose capsid is indistinguishable from those of HBV virions [14]. However, Cp149 particles do not incorporate any nucleic acid [15]. The last 34 residues of Cp183, i.e., the C-terminal domain (CTD), are rich in serines and arginines, and are responsible for interaction with RNA [16]. Phosphorylation of the serines, particularly S155, S162 and S172, is required for specific packaging of pgRNA•RT *in vivo* [17]–[19]. Phosphorylation status of Cp183 CTD was also found to be associated with intracellular transport of HBV cores. Only phosphorylated HBV cores reached the nucleus [20], [21] and only mature cores were imported into the nucleus [22]. Dephosphorylation was observed during HBV core maturation and correlates with subsequent envelopment and secretion [23]–[25].

We have studied Cp149 and Cp183 assembly *in vitro* [26]. Dimeric Cp149 is soluble and spontaneously assembles, in an entropically driven reaction, into *T* = 4 capsids as a function of protein concentration, ionic strength and temperature [27]. In contrast, dimeric Cp183 is not substantially soluble under physiological conditions. To control *in vitro* assembly, we used non-denaturing concentrations of guanidine hydrochloride (GuHCl) to keep Cp183 dimers in solution (Figure 1B). Decreasing the concentration of GuHCl induced capsid assembly along with precipitation of some Cp183. We speculated that for *in vivo* HBV core assembly to proceed in a regulated manner, a chaperone, instead of GuHCl, would be required to keep newly expressed Cp183 from precipitating, self-assembling, or assembling around random nucleic acid. There must also be a mechanism to release the chaperone to allow assembly when the right assembly nucleation center, RT-bound pgRNA, is available. Since Cp183 is phosphorylated prior to or during HBV core assembly [18], the phosphorylating kinase may well act as a non-canonical chaperone.

One of the kinases suggested to phosphorylate Cp183 *in vivo* is a member of the SR protein kinase (SRPK) family. SRPKs specifically phosphorylate serines within the arginine/serine repeats (RS domain) of an SR protein [28], [29]. SR proteins share a remarkable sequence similarity with the Cp183 CTD (Figure 1A) [30]. SR proteins are RNA-binding molecules that have roles in spliceosome positioning and RNA transport from the nucleus. SRPK1 and SRPK2 were co-purified with GST-tagged HBV core proteins from Huh-7 cell lysates, and they demonstrated kinase activity biochemically identical to HBV core kinase activity in the cell lysate [31]. Interestingly, SRPKs were also shown to influence HBV life cycle independent of kinase activity as overexpression of SRPK1 and SRPK2, even catalytically inactive mutants, suppressed HBV replication [32]. Though SRPK2 actually has the higher affinity for HBV [32], a truncated form of SRPK1 has been exhaustively characterized. SRPK1 binds to the typical substrate ASF/SF2 with a K_d_ ∼ 50 nM and functions with a processive phosphorylation mechanism [33], [34]_ENREF_25. The structure of SRPK1 comprises a small N-terminal lobe of primarily connected by a spacer region to a larger C-terminal lobe [35]. While the spacer is important for subcellular localization [36], the two lobes constitute the kinase core. A mutant lacking the spacer as well as part of the N-terminal lobe, SRPK1ΔN1S1, has been shown to maintain substrate specificity and kinase functionality *in vitro* [33], [34].

In this article, we discuss *in vitro* studies of Cp183 interaction with SRPK1ΔN1S1 (abbreviated as SRPKΔ in the rest of the article). Using a column-based binding assay, we showed that SRPKΔ bound to Cp183 at the CTD. When SRPKΔ bound to Cp183 dimers, the core protein was unable to self-assemble; assembly was subsequently reactivated when ATP-induced phosphorylation decreased the stability of the SRPK/Cp183 complex. Thus, we demonstrated a kinase-gated mechanism of HBV assembly where the kinase served as a non-canonical chaperone. SRPKΔ also bound to Cp183 capsid. We established a centrifugation-based titration assay to show the stoichiometry to be 49±3 SRPKΔ per capsid. Image reconstructions of cryo-EM data identified 30 multivalent SRPKΔ-binding sites at the capsid twofold vertices. These sites coincide with pores in the capsid that are proximal to the core protein CTDs. These observations indicate that the CTD is transiently exposed to the capsid exterior, possibly by threading through the pores. However, SRPKΔ did not bind to RNA-filled capsid, implying tunable accessibility of Cp183 CTDs depending on nucleic acid-capsid interaction. We suggest that nucleic acid-sensitive exposure of the CTDs provides a mechanism for directing the intracellular transport of HBV.

## Results

### SRPKΔ binding to HBV capsids requires the core protein CTD

As a qualitative assay, we tested capsids for their ability to bind His-tagged SRPKΔ adsorbed onto a Ni^++^-column. Three types of capsid were assayed: empty reassembled Cp183 capsid, empty reassembled Cp149 capsid [14]_ENREF_6 and Cp183 capsid filled with heterogeneous RNA from the expression system [37]_ENREF_1 (see Supporting Figure S1).

Cp183 associated with column-bound SRPKΔ. Without SRPKΔ pre-loaded to the Ni^++^-column, empty Cp183 capsid flowed through the column freely; however, a substantial fraction of empty Cp183 capsid bound to the SRPKΔ-loaded column and co-eluted with SRPKΔ, indicating interaction between the capsid and SRPKΔ. The earlier fractions of the eluate were richer in Cp183 than later ones, implying that binding of capsid weakened the interaction of the His-tagged SRPKΔ with the Ni^++^-column. Moreover, it was observed that more Cp183 capsid bound to the column when the flow rate was slowed from 0.5 ml/min to 0.3 ml/min or when the salt concentration was decreased from 0.5 M to 0.3 M (data not shown). The former observation showed that capsids bind SRPKΔ with relatively slow binding kinetics, with a half-time on the order of minutes. The latter observation suggested that the interaction between SRPKΔ and Cp183 capsid is electrostatic in nature.

In contrast to empty Cp183 capsids, both Cp149 capsids and RNA-filled Cp183 capsids ran through the Ni^++^-column freely with or without bound SRPKΔ. Cp149 is a core protein mutant lacking the serine and arginine-rich CTD of Cp183. Its failure to bind to the SRPKΔ-loaded column was consistent with our assumption that the SR protein-like CTD is the substrate for SRPK. In the case of RNA-filled Cp183 capsids, the interaction to RNA probably traps the basic CTD inside and prevents its interaction with the external SRPKΔ.

These studies raise the question of CTD accessibility on the capsid exterior. Structural studies [38], [39] and the internal location of packaged nucleic acid imply that the CTDs are on the interior of a Cp183 capsid. In order for it to be accessible to column-adsorbed SRPKΔ, we are led to hypothesize that the CTD must at least transiently penetrate through the capsid. Thus, the ionic strength dependence of binding may also reflect a change in capsid stability.

### Multiple SRPKΔ molecules bind to a Cp183 capsid

To measure the binding stoichiometry and affinity between SRPKΔ and Cp183 capsid, we titrated empty reassembled Cp183 capsid with SRPKΔ. The titration was to be plotted as *n*, the average number of SRPKΔ per capsid, versus [S], the concentration of unbound SRPKΔ. In the simplest model, all binding sites on a capsid are equivalent and independent. The maximum number of binding sites per capsid is *N* and the microscopic dissociation constant is *K*_D_. The relationship between *n* and [S] should lead to a hyperbolic curve 
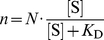
(1)


Experimentally, we sedimented the capsids along with bound SRPKΔ, and measured the concentration of SRPKΔ remaining in the supernatant, [S]_A_, using densitometry of SDS-PAGE. In our data sets, [S]_A_ did not go beyond 200 nM, because of protein precipitation at higher concentrations. To make sure that titration data reflected binding to capsids, we also monitored reactions using dynamic light scattering (data not shown). For [S]_A_<200 nM, the light scattering always indicated a single dominant species of ∼ 40 nm, the hydrodynamic size of a Cp183 capsid. The scattering intensity grew with increasing [S]_A_ due to deposition of SRPKΔ on the capsids. As light scattering is particularly sensitive to large complexes, this result does not exclude the presence of dimer. As [S]_A_ went above 200 nM, the peak broadened and shifted to a larger size, suggesting aggregation and polydispersity; eventually a protein precipitate was visible.

The average number of SRPKΔ per capsid was calculated according to 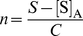
(2)in which *C* and *S* were input concentrations of capsid and SRPKΔ respectively.

**Figure 2:**
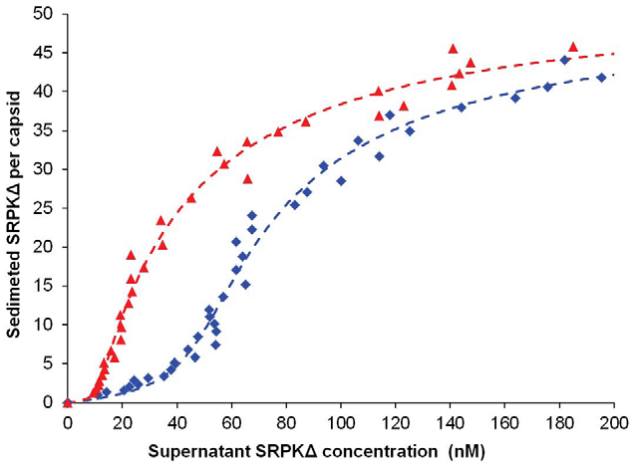
Titration of Cp183 capsid by SRPKΔ. Titrations were performed to determine the binding constant and stoichiometry of SRPKΔ for Cp183 capsids and the binding constant of SRPKΔ for Cp183 dimer. SRPKΔ was added to Cp183 assembly reactions, a mixture of capsids and residual Cp183 dimers (blue curve), or Cp183 assembly reactions that had been depleted of free dimers by SEC (red curve). Mixtures were incubated overnight at 4°C and then centrifuged to pellet capsid and bound SRPKΔ; SRPKΔ remaining in the supernatant was determined by densitometry of SDS-PAGE. Data are reported as the number of sedimented SRPKΔ molecules per capsid. Theoretical curves were fit to the data assuming equivalent non-interacting sites on capsids or dimers. On average, there were 49±3 SRPKΔ molecules per capsid binding with 31±3 nM dissociation constant and dissociation constant of 0.6±0.4 nM for each C-terminus on a free dimer.

While we had expected hyperbolic binding isotherms for *n* vs [S]_A_, assuming [S] = [S]_A_, the binding curves turned out to be sigmoidal (Figure 2, blue curve). A likely explanation was contamination of Cp183 capsid with a small amount of Cp183 dimer. A free dimer with a pair of fully exposed CTDs could bind SRPKΔ to form small complexes that did not co-sediment with capsid. Cp183 dimer has such a poor solubility in the absence of GuHCl that we do not typically remove dimer from in vitro assembled Cp183 capsid. However, a residual amount at the nM level could remain after the capsid reassembly reaction or arise from dissociation of Cp183 capsids. To test this hypothesis, we purified capsid by size exclusion chromatography (SEC) prior to SRPKΔ titration and found that the initial lag phase in the titration curve was substantially reduced (Figure 2, red curve).

In light of the dimer-SRPKΔ side reaction, the binding model can be described as
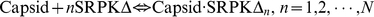
(3)

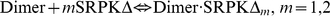
(4).

Correspondingly, the simulation equation for the titration curves is modified from equation 1 to equation 5: 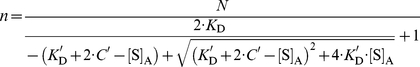
(5)where *K*_D_ denotes the dissociation constant of each CTD on a free dimer and *C'* is the free dimer concentration (a derivation of Equation 5 is provided in Supporting Text S1).

Equation (5) fits the titration data well (Figure 2 and Table 1). Based on four independent experiments and curve fits, there are 49±3 equivalent and non-interacting SRPKΔ binding sites on a capsid. SRPK binds each site with a dissociation constant of 31±3 nM. This value is similar to that of the interaction between SRPKΔ and a typical substrate SR protein, e.g. 50 nM for ASF/SF2 [33]. Remarkably, the dissociation constant of SRPKΔ for free Cp183 dimer is 0.6±0.4 nM, an affinity almost two orders of magnitude stronger, suggesting that Cp183 has evolved to mimic an ideal SRPK substrate.

**Table 1:** Curve fits for titrations of Cp183 capsid by SRPKΔ.

	A	B	C	D	Average
*_N_*	51.17	45.35	47.97	52.84	49±3
*K*_D_ (nM)	32.03	32.39	27.68	33.40	31±3
*K*_D_'(nM)	1.21	0.40	0.54	0.25	0.6±0.4
*C*' (nM)	25.09	7.78	18.54	5.71	

There were 4 trials of titration: A, B, C and D. In trials B and D, the free dimer concentration (*C*') was depleted by size exclusion chromatography prior to the titration.

### SRPKΔ binds to the exterior of Cp183 capsids

To examine how SRPKΔ was able to bind the CTD, which is localized to the interior of a capsid, we determined the structure of Cp183 capsid saturated with SRPKΔ. Electron micrographs of frozen-hydrated Cp183 capsids revealed empty spherical particles with a diameter of 32 nm (Supporting Figure S2).

**Figure 3:**
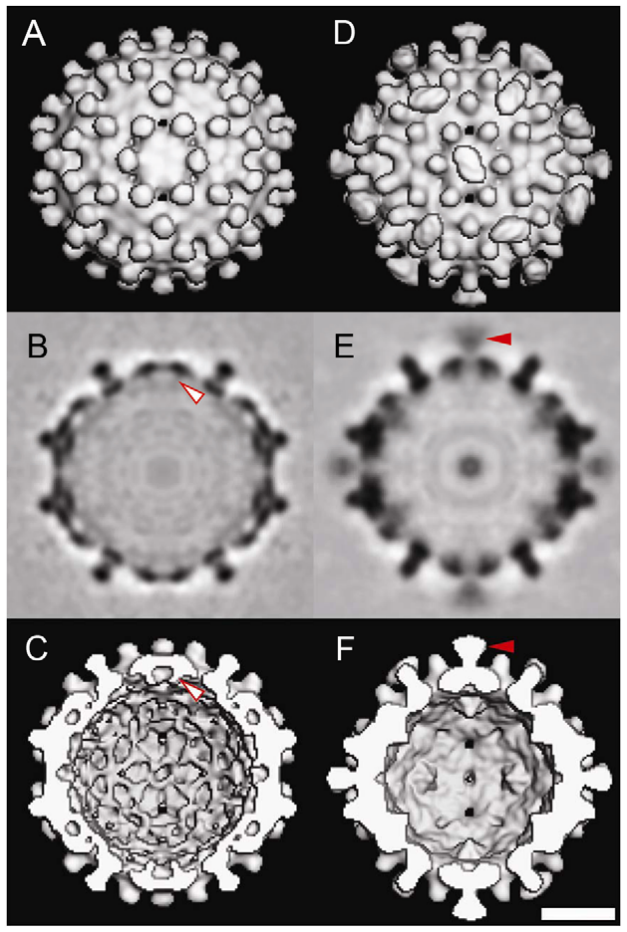
Three-dimensional structures of Cp183 Capsid and Cp183 Capsid/SRPKΔ. Cp183 capsid (A, B, C) and capsid/SRPKΔ (D, E, F) are displayed as exterior views (A, D), gray scale central sections (B, E), and interior views (C, F). Surface-shaded representations were contoured to account for 100% of the expected protein volume for a Cp183 capsid. The closed red arrow identifies the external SRPKΔ density at twofold vertices; the open red arrow highlights that the interior density attributable to the CTDs. Binding of SRPKΔ correlates with remodeling the capsid interior (compare B, C with E, F) resulting in the loss of CTD density under twofold vertices. All views are along an icosahedral twofold axis. The scale bar represents 10 nm.

To examine the structure of Cp183 without modification by SRPK, we determined the structure of empty Cp183 to 1.7 nm resolution. The 3-D image reconstruction of the empty Cp183 capsid showed an overall architecture that is similar to that of both Cp149 capsid [40] and Cp183 capsid filled with a heterogeneous mixture of RNA [38]. These particles have a *T* = 4 surface arrangement with 30 two-fold (quasi-sixfold) vertices; the 120 spikes projecting from the capsid surface are the four-helix bundles of the dimer interface (Figure 3A–C). Unlike Cp149 capsids, the outer surface of the control Cp183 structure showed no openings at icosahedral twofold and fivefold vertices (Figure 3A). In the Cp183 capsid (Figure 3B and 3C), extra density was observed within the capsid interior crossing the icosahedral twofold (quasi-sixfold) vertices. This density, and corresponding density under the icosahedral fivefolds, is distinct from the relatively smooth inner surface observed in Cp149 capsids. We attribute this internal density to the icosahedrally averaged CTDs, as they are expected to extend from the contiguous shell near these vertices [38], [39].

Similar to Cp183 capsid, Cp183 capsid/SRPKΔ, calculated to 1.4 nm resolution, has a diameter of 32 nm for the contiguous surface (Supporting Figure S2B) and *T* = 4 icosahedral symmetry. However, it exhibits 30 exterior funnel-shaped units of density connecting to the capsid at each icosahedral twofold vertex (Figure 3D–E). To gain an understanding of the geometry and occupancy of this additional density, the 3-D density map was rendered assuming the presence of 240 Cp183 core proteins assuming an average protein density of 1.36 g/cm^3^. The SRPKΔ density extended about 4.1 nm from capsid surface, and the funnel top was about 2.8 nm by 3.8 nm. In the central section of the Cp183 capsid/SRPKΔ reconstruction (Figure 3E), it is clear that this new density is weaker than adjacent capsid density. We also note that the SRPK density was substantially stronger at lower resolution (data not shown), suggesting conformational heterogeneity. Comparable SRPKΔ density was also observed in Cp183 reconstructions with lower concentrations of SRPKΔ (Supporting Figure S3).

On the interior, in contrast to empty Cp183 capsids, CTD density under the icosahedral twofold vertex of Cp183 capsid/SRPKΔ was extensively remodeled (Figure 3). This is especially evident in the central section of the reconstructions. In these images (Figure 3B, E), Cp183 CTD twofold density is absent from Cp183 capsid/SRPKΔ.

**Figure 4:**
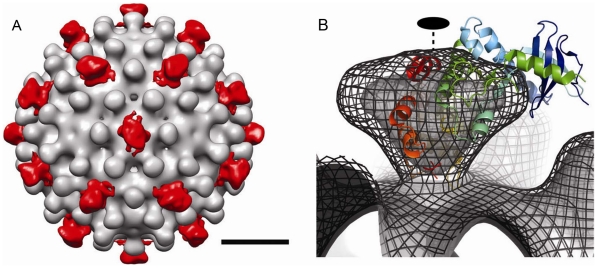
The difference map and modeling of SRPKΔ on Cp183 Capsid. (A) To isolate the density attributable to SRPKΔ, a difference map of SRPKΔ (red color) was calculated by subtracting Cp183 Capsid from Cp183 capsid/SRPKΔ and superimposed on the corresponding region of the 3-D reconstruction of Cp183 Capsid. The resulting densities of SRPKΔ were found located at each twofold axis. The bar represents 10 nm. (B) Cryo-EM density of Cp183 capsid/SRPKΔ fitted with SRPKΔ coordinates (as shown in the cartoon representation) viewed at twofold position. The 3-D reconstructions rendered in isosurface and isomesh modes represent 100% contour and a 1 σ contour, respectively. The fitting was performed manually to place the SRPKΔ active site close to the twofold pore and maximize envelopment of the large lobe of SRPKΔ in cryo-EM density. The twofold axis is marked with a dashed line.

To examine the external density attributed to SRPKΔ, we calculated a difference map by subtracting the Cp183 map from that of Cp183 capsid/SRPKΔ (Figure 4). In this map, SRPKΔ appeared to be bound above the icosahedral twofold vertex. The cryo-EM density map could only fit part of the substrate binding C-terminal lobe of the SRPKΔ atomic structure (PDB accession code: 1WBP) [34]. At lower contour levels, the large lobe could be fully covered (data not shown). The weakness of the electron density and its inability to account for the volume of the molecule, suggested that the SRPKΔ position is variable and non-icosahedral.

### SRPKΔ gates Cp183 capsid assembly

The binding data was consistent with the hypothesis that SRPKΔ (or a similar kinase) does act as a non-canonical chaperone, preventing assembly when bound to dimer. We observed that SRPKΔ had a higher affinity for dimer than capsid, suggesting that SRPKΔ binding should favor the dissociated state. Furthermore, crowding by SRPKΔ at twofold vertices was also expected to disfavor assembly. The missing catalyst for an assembly reaction is a mechanism to release bound SRPKΔ, activating assembly.

**Figure 5:**
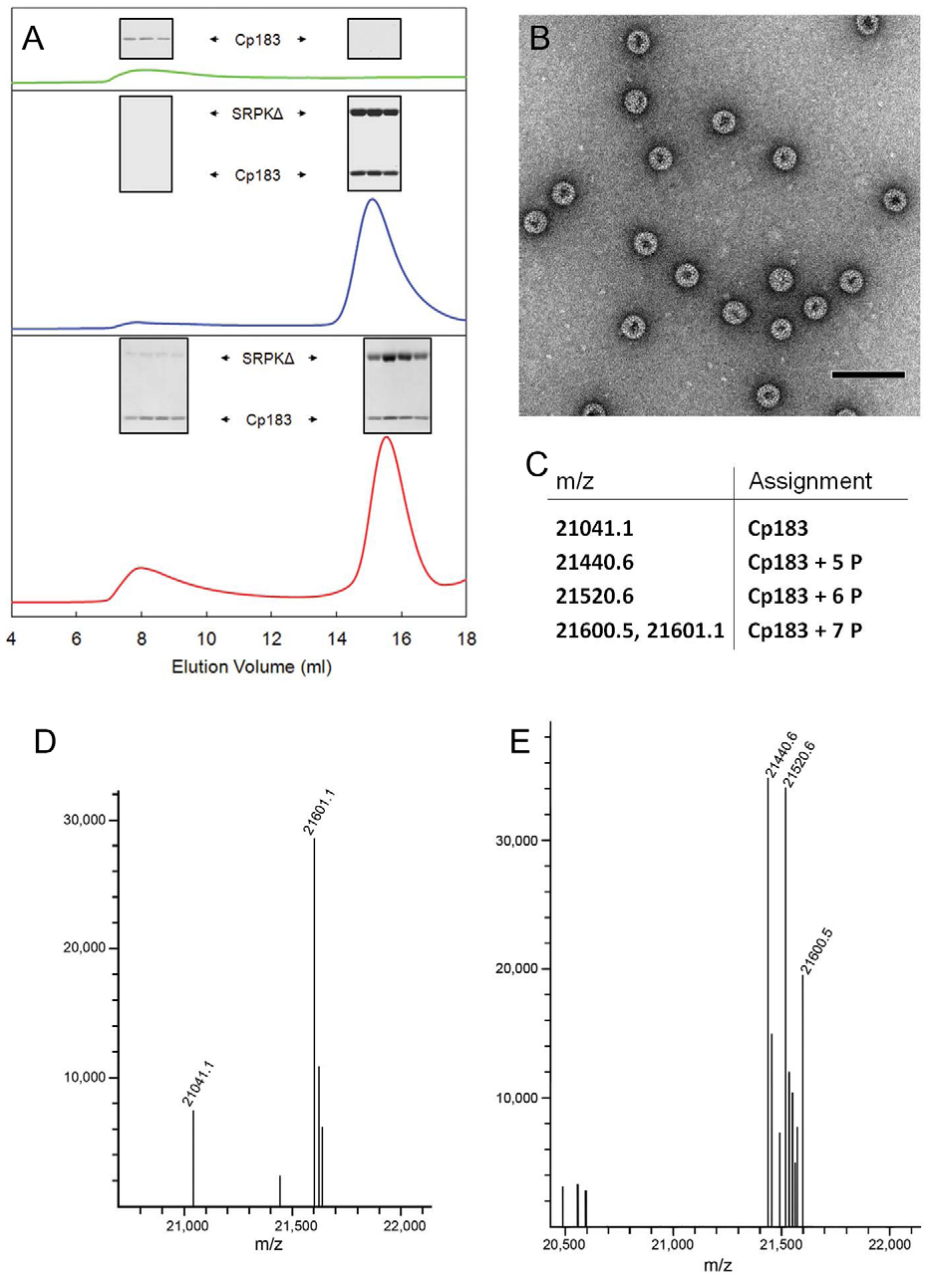
*In vitro* SRPKΔ-gated HBV capsid assembly. SRPKΔ was used as an *in vitro* chaperone to prevent Cp183 aggregation and regulate Cp183 self-assembly. (A) Size exclusion chromatographs of Cp183 capsid assembly products. The insets show SDS-PAGE of the indicated SEC fractions. Cp183 capsid assembly was normally induced by dialyzing GuHCl from Cp183 dimer solution, resulting in reassembled capsid eluting at ∼8 ml and a significant loss of Cp183 to precipitation (green). Mixing SRPKΔ and Cp183 dimer prior to dialysis prevented aggregation or capsid assembly; instead a stable soluble complex formed and eluted at ∼15 ml (blue). Subsequent dialysis of the Cp183 capsid/SRPKΔ complex against ATP/Mg^++^ resulted in a mixture of capsid, complex, and presumably free SRPKΔ (red). (B) Negative stained TEM of the capsid fraction from an SRPKΔ-gated Cp183 assembly reaction shows morphologically normal HBV capsids. The majority of the particles are ∼35 nm in diameter with a minor ∼30 nm diameter population. The scale bar is 100 nm. (C) Table of species identified in the mass spectra shown in panels d and e. (D) MS of the capsid fraction from an SRPKΔ-gated assembly reaction. Most of the Cp183 from the capsid fraction was phosphorylated at seven sites, with a relatively small portion of unphosphorylated and partially phosphorylated protein. (E) MS of the lower molecular weight complex fraction after SRPKΔ-gated assembly. Most Cp183 was phosphorylated at 5 to 7 sites; the relatively large fraction of partially phosphorylated Cp183 in this pool suggests that it is more likely to remain bound to SRPKΔ.

To test the hypothesis that SRPK1 could prevent self-assembly, we examined the effect of SRPKΔ on *in vitro* assembly (Figure 5). Typically, to drive *in vitro* assembly of empty Cp183 capsids, a solution of Cp183 dimer, solubilized in non-denaturing concentrations of GuHCl, was dialyzed against a GuHCl-free buffer (reassembly buffer) [26]. Using a reassembly buffer of 0.25 M in ionic strength, a substantial amount of Cp183 precipitated, while the rest assembled into capsid as shown by SEC. In comparison, dialysis of a mixture of a 1∶2 molar ratio of Cp183 dimer and SRPKΔ in 0.5 M GuHCl against the reassembly buffer resulted in a soluble mixture in which Cp183 did not precipitate or assemble. Cp183 and SRPKΔ co-migrated as a single peak eluting earlier than either SRPKΔ or Cp183 dimer (data not shown), indicating that the two proteins form a stable complex, presumably dimer•SRPKΔ or dimer•SRPKΔ_2_ or both. SDS-PAGE of the co-migration peak also indicated more than one SRPKΔ per Cp183 dimer (Figure 5A). Thus, these experiments showed that SRPKΔ acts to solubilize Cp183 dimer and inhibits its assembly.

To be biologically relevant, this reaction pathway should include an assembly-reactivation mechanism to remove the SRPKΔ protecting group. We reasoned that, like most kinases, SRPKΔ would have a much lower affinity for phosphorylated substrate. Therefore, we dialyzed Cp183/SRPKΔ complex against a solution of ATP/Mg^++^ to allow phosphorylation of Cp183. Following dialysis, SEC indicated capsid formation as well as a large fraction of Cp183 remaining in the complex. The assignment of the capsid peak was confirmed by electron microscopy, which showed ∼35 nm diameter particles typical for *T* = 4 HBV in negative stain EM [5]. Like all core protein assembly reactions, there was also a small population of ∼30 nm diameter particles, presumably with *T* = 3 symmetry [41]. SDS-PAGE of the capsid peak indicated a much smaller proportion of SRPKΔ in reassembled capsid than in the Cp183/SRPKΔ complex, confirming that dissociation of SRPKΔ allows assembly.

SRPKΔ was expected to phosphorylate Cp183 during ATP-gated assembly. Based on ESI-MS, the majority of Cp183 in the capsid peak was decorated with seven phosphate groups (Figure 5D). A smaller pool of Cp183 was unphosphorylated, and a minor population of Cp183 had five phosphates. Unassembled Cp183 that co-eluted with SRPKΔ also acquired five or more phosphates (Figure 5E). These observations indicate that even after phosphorylation, SRPKΔ can remain associated with Cp183, though with reduced affinity. From Le Chatelier's principle we predict that gradual release of phosphorylated Cp183 will lead to further capsid assembly. Indeed, phosphorylated Cp183-SRPKΔ fractions slowly assembled into EM-observable capsids over a few days.

## Discussion

In our *in vitro* experiments, we tested hypothetical regulatory mechanisms of the HBV lifecycle by employing SRPKΔ for unconventional functions: (i) a probe that labels the CTD exposed on a HBV capsid, demonstrating that the CTD location is dynamic; (ii) a non-canonical chaperone that gates HBV capsid assembly. SRPK1 or SRPK2 affect Cp183 phosphorylation and assembly in vivo [42], [43].

SRPKΔ binds to Cp183 at the CTD (Figure 3); truncation of the core protein or engaging the CTD with RNA eliminates the SRPKΔ-capsid interaction (Supporting Figure S1). In capsids, the CTDs are localized to the capsid interior, extending from the assembly domain of the core protein near the pores at capsid twofold and fivefold vertices [12], [39]. There are potentially 240 SRPKΔ-binding CTDs per *T* = 4 capsid. However, titration of Cp183 capsid by SRPKΔ fits a model of 49±3 equivalent and independent SRPKΔ binding sites per capsid (Figure 2, Table 1). Image reconstructions only show 30 units of relatively weak SRPKΔ density on the capsid exterior at the twofold vertices (Figure 3 and 4). The number difference between solution experiments and the reconstruction is most likely due to multiple binding at each of the 30 twofold vertices, each of which has six CTDs. Because of increasing steric hindrance, the binding constants at each site probably decrease as more SRPKΔs bind. The curve fit describes the simplest model consistent with the binding data; a more complex model that includes multiple binding constants for multiple SRPKΔs per vertex could easily fit but would be inconclusive. Nonetheless, the binding data do indicate that at least two SRPKΔ molecules can bind at each twofold. The weakness of the SRPKΔ density is attributable to disorder: there are six non-equivalent CTDs around a twofold vertex, each of which carries seven phosphorylation sites (Figure 1) and is likely to be very flexible. After 60-fold averaging, the large volume that could be occupied by one or more bound SRPKΔs is represented by a small high occupancy core (Figure 4).

Binding of SRPKΔ to the CTDs of a capsid requires exposure of the CTDs to the outer surface, at least transiently, since SRPKΔ is too big (>2.9 nm in any dimension) [34] to fit through the capsid pores (<1.5 nm at twofold axes and ∼ 0.3 nm at fivefold axes) [39]. Transient exposure of CTDs has been previously suggested to allow additional interactions with host machinery to regulate the HBV life cycle [44]. This hypothesis is supported by correlations that associate CTD phosphorylation with intracellular trafficking [20], [23]–[25]. As a more direct observation, this paper visually demonstrates the CTD exposure and its interaction with a host protein.

**Figure 6:**
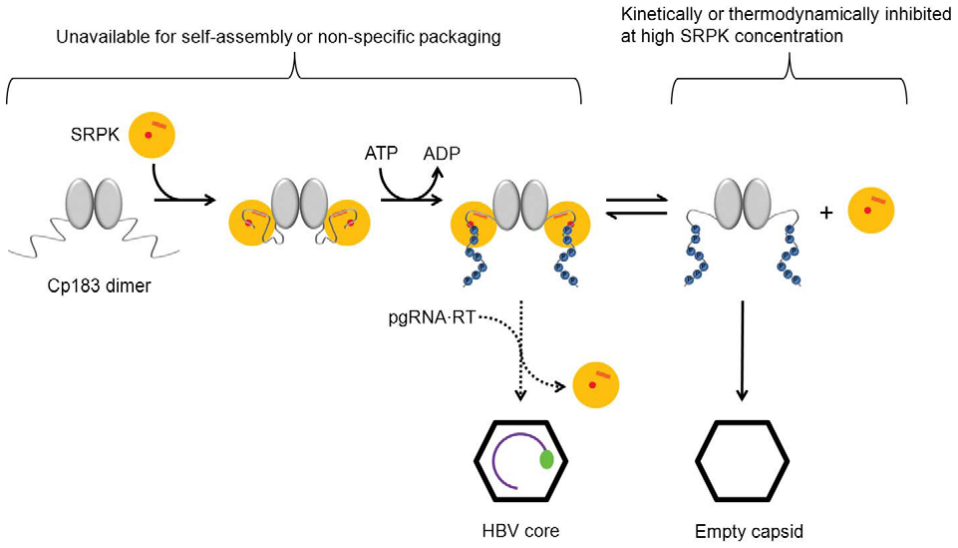
Scheme describing SRPK-gated mechanism of HBV core and capsid assembly. Binding of SRPK to unphosphorylated Cp183 prevents self-assembly and packaging nucleic acid. Subsequent phosphorylation weakens SRPK association to slowly release free Cp183 to form capsid. Even modest concentrations of free SRPK should suppress the concentration of free Cp183 and therefore prevent capsid assembly. In vivo, a likely catalyst of Cp183 release is reverse transcriptase-bound pgRNA that can displace weakly bound SRPK and induces assembly resulting in packaging specificity.

Furthermore, our study suggests a mechanism for the regulation of CTD exposure and accessibility which can signal core maturation (Figure 6). We observed that in empty capsids the CTDs were able to bind SRPKΔ (Supporting Figure S1). However, if the capsid was filled with RNA, no binding was observed. Our interpretation is that the negatively charged RNA retains the basic CTDs inside the capsid and prevents externalization. In the context of the HBV life cycle, a control mechanism is necessary to distinguish mature cores from immature ones; we suggest this is mechanism is based on exposure of the CTD. Only mature HBV cores are enveloped and secreted [11], [12] or transported to the nucleus [22]. We speculate that reverse transcription, which occurs within the HBV core, allows exposure of CTDs. The interaction between CTDs and single stranded RNA, which is very flexible and can contort to interact with all CTDs, is able to restrict CTD exposure. The partially double stranded DNA genome of the mature core is expected to be much less flexible and much less able to engage CTDs; thus, reverse transcription will allow exposure of at least some of the 240 CTDs in each capsid. In this paper we have shown that RNA-filled capsids do not appreciably bind SRPKΔ. Similarly, it was observed that RNA-filled capsids are not appreciably phosphorylated by exogenous protein kinase C unless they are partially disassembled, whereas kinase activity that co-purifies with virions is able to add two to four phosphates per virion (as ^32^P), indicating the availability of only one to four CTDs out of the 240 in a capsid [45]. A maturation-dependent change in CTD accessibility would allow cores to bind proteins such as SRPK or importin α/β to direct trafficking [20]–[22]. Accessibility of the CTD is likely, as it is the CTD that carries the HBV core nuclear localization signal [46], [47].

There are two explanations for the ability of the CTD to be exposed in our experiments: breathing/partial opening of the capsid or exit of the CTD through the pre-existing hole on the twofold. We find that breathing alone is inadequate to explain CTD exposure. It has been proposed that capsid dynamics, breathing modes, can facilitate CTD exposure [44]. Breathing modes have been shown to expose buried and internal peptide segments in flock house virus [48], rhinovirus [49], and HBV [44]. In HBV, breathing modes appear to involve a partial unfolding of the core protein near the C-terminus of the assembly domain, exposing a buried residue to proteolytic digestion. The unfolding equilibrium constants for Cp149 capsid between 19°C and 37°C are documented [44]. By extrapolation, we can obtain the value for our experimental condition, 4°C and it is 6×10^−5^. This number tells the chance for a CTD to become externalized through the breathing mode. In comparison, our titration data provide an experimental value for CTD exposure rate if we attribute the affinity difference between capsid/SRPKΔ and dimer/SRPKΔ to the availability of the core protein CTDs. The dissociation constant for capsid/SRPKΔ and dimer/SRPKΔ are 31 nM and 0.6 nM, respectively; hence the exposure rate of a CTD at a twofold vertex is 0.6/31 = 0.02. As there are 6 CTDs around a twofold axis, the exposure rate contributed by each CTD is about 0.02/6 = 0.003. This value is two orders of magnitude higher than the unfolding rate of a Cp149 capsid. The discrepancy may reflect that Cp183 is more labile than Cp149, or indicate that the highly flexible CTDs can simply thread through the large capsid pore at a twofold vertex without involving a breathing mode.

We have demonstrated a SRPKΔ-gated capsid assembly mechanism *in vitro*. *In vivo*, a different protein may serve as a chaperone to minimize Cp183 self-assembly at an inappropriate time. However, SRPK1 and SRPK2 are particularly attractive candidates for the regulatory chaperone, as it can be released by phosphorylation, resulting in an assembly reactivation mechanism (though we cannot exclude other kinases with high affinity for substrate). It has been previously observed that SRPK2 co-immunoprecipitates with HBV core protein in the context of huh7 cells [42]. Similarly, overexpression of either SRPK1 or SRPK2 inhibits replication of HBV, with the stronger inhibitory effect associated with SRPK2 [43]. We note that phosphorylation does not cause SRPKΔ to release Cp183; it weakens a very strong association. Some of the phosphorylated Cp183 did not proceed to self-assembly immediately; rather, it stayed bound to SRPKΔ in a soluble complex. Thus, even after phosphorylation, SRPKΔ retains a useful chaperone activity (Figure 6). The observation of continued assembly of the SEC-purified phosphorylated Cp183/SRPKΔ complex over several days supports this hypothesis. In the presence of excess SRPK mass action would favor the persistence of the phosphorylated Cp183/SRPK complex. Consequently self-assembly of Cp183 would be kinetically and thermodynamically inhibited. Assembly activation would require a specific high affinity nucleating complex to displace weakly associated SRPK and initiate assembly. *In vivo*, the pgRNA•RT complex may serve this role.

## Materials and Methods

### Cp183 dimer and capsids

Cp183 capsids filled with host RNA were harvested from an *E. Coli* expression system [37]. Cp183 dimers were purified as previously described [26]. Briefly, capsids were destabilized in 1.5 M guanidine, 0.5 M LiCl, 10 mM DTT, and 20 mM Tris-HCl at pH 7.4 (disassembly buffer). In disassembly buffer, most RNA was sedimented with Li^+^ and the residual amount was separated from Cp183 dimer by SEC. The purified Cp183 dimer was stored in disassembly buffer and the concentration was determined by UV absorbance (ε_280_ = 60,900 M^−1^•cm^−1^) [50].

To generate empty Cp183 capsids, Cp183 dimer was dialyzed against 0.25 M NaCl, 10 mM DTT and 20 mM Tris-HCl at pH 7.4 (reassembly buffer) [26]. Some samples of reassembled capsid were further purified from residual free dimer using a Superpose 6 column. Capsid concentration was measured by scattering-corrected UV absorbance as previously described [50]. When necessary, purified capsids were concentrated by adsorption to a Mono-Q column, from which they were eluted at by ∼0.5 M NaCl. Reassembled empty capsids were stored at −80°C in reassembly buffer with 30% glycerol. To validate the integrity of stored Cp183 capsids they were examined by SDS-PAGE, EM, dynamic light scattering, and affinity towards SRPKÄ. Stored Cp183 capsids showed no evidence of proteolytic degradation by SDS-PAGE. By negative stain EM and cryo-EM particle morphology remained consistent with numerous previously published micrographs (see Supporting Figures S 2). The diameter of stored capsid (ca 40 nm), by dynamic light scattering, was the same as freshly prepared capsid, indicating minimal aggregation. Both freshly prepared and stored Cp183 capsids showed high affinity for SRPKΔ (experiments shown in Supporting Figure S1 used fresh capsids, experiments shown in Figure 2 used stored capsids).

### SRPKΔ preparation

A plasmid coding SRPKΔ was a gift from Dr Gourisankar Ghosh (UCSD). Protein expression and purification through a His-Trap column has been described [33]. For further purification, the eluate from the His-Trap column was loaded onto a Mono-Q column, from which SRPKΔ was eluted at 0.2 M NaCl. The protein concentration was calculated from UV absorbance. The extinction coefficient, ε_280_ = 74,745 M^−1^•cm^−1^, was determined using the Edelhoch method [51] and confirmed by cysteine reaction with dithionitrobenzoic acid. When necessary, purified SRPKΔ was concentrated using a His-Trap Column.

### SRPKΔ-capsid binding assay on a His-Trap column

For these experiments, purified capsids (reassembled Cp183 capsids, reassembled Cp149 capsids [14] and Cp183 capsids filled with *E. Coli* RNA [37]) and SRPKΔ were all exchanged into 20 mM imidazole, 0.3 M NaCl and 20 mM phosphate at pH 7.4 (buffer A). Samples of SRPKÄ were adsorbed onto a 1 ml His-Trap column and the column was equilibrated with buffer A at 4°C. A capsid sample (0.1 ml) was then loaded on the column, followed by a programmed elution using 5 ml of buffer A, 1 ml of gradient change from buffer A to buffer B (0.5 M imidazole, 0.3 M NaCl and 20 mM phosphate at pH 7.4) and 5 ml of 100% buffer B. Control runs were executed by replacing either SRPKΔ or the capsid sample for an equal volume of buffer A. Fractions from each run were tested by SDS-PAGE.

### Titration of Cp183 capsid by SRPK

Purified SRPKΔ and Cp183 capsids were exchanged into 0.30 M NaCl, 10 mM DTT and 20 mM Tris-HCl at pH 7.4 (buffer R). A series of 150 µl reactions, consisting of Cp183 capsid (4 µM dimer concentration) and SRPKΔ ranging from 60 nM to 2 µM all in buffer R, were incubated overnight at 4°C in BECKMAN Polycarbonate Centrifuge Tubes. The tubes were then centrifuged in Optima™ MAX-XP Ultracentrifuge (BECKMAN COULTER) at 4°C and 150,000 g for half an hour. Under this condition, >95% of the capsids sedimented while >95% of free SRPKΔ stayed in the supernatant. To determine the amount of SRPKΔ remaining in solution after centrifugation, supernatants and SRPKΔ concentration standards were loaded onto 10% SDS-PAGE. The gels were silver stained and the densities of bands in scanned gels were quantified using ImageJ.

### Electron microscopy and image processing

SRPKΔ-decorated Cp183 capsid was prepared by mixing 5.6 µM SRPKΔ and 5.8 µM (dimer concentration) Cp183 capsid in 0.53 M NaCl, 10 mM DTT and 20 mM Tris-HCl at pH 7.4. The reaction was incubated at 4°C for 4 days prior to cryo-EM.

Specimens for electron cryo-EM were vitrified and imaged by the well established procedures as previously described [52]. Briefly, a 3.5 ìl drop of sample was applied to a glow-discharged holey carbon-coated grid (Quantifoil R2/2). The grid was then plunged into liquid ethane cooled by liquid nitrogen using an FEI Vitrobot™. All subsequent steps were carried out with the specimens kept below −170°C to avoid the devitrification. The grid was transferred to a Gatan 626DH cryo-holder (Gatan Inc., USA), and examined in a JEM-3200FS electron microscopy (JEOL Ltd., Japan) operated at 300 kV. Images were recorded at multiple defocuses on a Gatan UltraScanTM 4000 4k x 4k CCD camera at a magnification of 80,000x for Cp183 capsid and 40,000x for Cp183 capsid/SRPKΔ under low-dose condition (≤14 e-/Å^2^). The pixel size was 0.1484 nm for capsid and for 0.2940 nm for capsid-SRPKΔ.

Selected images with minimum astigmatism and drift were processed using EMAN2 (v 2.0) [53] and AUTO3DEM (v 3.15) software packages [54]. Particles were semi-automatically picked using e2boxer.py. The initial 3-D model was generated using the ab initio random model reconstruction method implemented in AUTO3DEM [54]. Origin and orientation searches were carried out iteratively using PPFT and further refined by PO2R [55]. The final 3-D maps of Cp183 capsid and Cp183 capsid/SRPKΔ were computed from 955 and 4399 particles using P3DR, respectively. The estimated resolution for Cp183 capsid was 17.4 Å and for Cp183 capsid/SRPKΔ was 14.2 Å using Fourier shell correlation at 0.5, calculated in EMAN2, as the criteria (Supporting Figure S4). Reconstructions were visualized using Robem, Chimera [56] and PyMOL [57].

To calculate a difference map, subtracting Cp183 capsid from Cp183 capsid/SRPKΔ, the region from radius 12.5–16.0 nm was used to scale the magnification and density. There was no detectable difference in the diameters of the capsids in the two reconstructions. In the resulting difference map, the solvent density was set to zero for radii smaller than inner surface (radius 11.2 nm) and for radii beyond the tip of the funnel-shaped density (radius 20.3 nm) (Figure 4).

### *In vitro* assay of SRPKÄ-gated HBV capsid assembly

Cp183 capsid assembly was set up in three ways for comparison: (i) 5.3 µM of Cp183 dimer in disassembly buffer was dialyzed overnight against reassembly buffer. (ii) 5.3 µM of Cp183 dimer was mixed with 11.2 µM of SRPKÄ in disassembly buffer and together they were dialyzed overnight against the reassembly buffer. (iii) The product from (ii), presumably a complex of Cp183 dimer with two SRPK molecules, was dialyzed against reassembly buffer plus 10 mM Mg^2+^ and 0.5 mM ATP. The reaction products were resolved by SEC, using a Superose 6 column, and the fractions were tested using SDS-PAGE.

### Accession codes

The SRPKΔ and Cp149 atomic structures and sequences are available from the protein data bank (PDB accession codes: 1WBP and 2G33, respectively) [34], [58]. Cp183 adds 34 C-terminal residues to Cp149; the Swiss Protein database accession code is P03147.1. The cryo-EM density maps of *T* = 4 HBV Cp183 capsid and Cp183 capsid/SRPKΔ have been deposited to EMDataBank.org. The EMDB accession codes are EMD-1969 and EMD-1968 respectively.

## Supporting Information

Figure S1 **Capsid binding to column-immobilized SRPKΔ.** (A) Binding assays to show that empty Cp183 capsids interact with SRPKΔ, in contrast to empty Cp149 capsids and RNA-filled Cp183 capsids. We preloaded His-tagged SRPKΔ onto a His-trap (GE Health Sciences) column, and then ran a capsid solution through. Bound protein was eluted (solid curves) with an imidazole gradient (pink curve); fractions were evaluated by SDS-PAGE (insets). As a control, binding of capsid and SRPKΔ were examined separately, and the chromatograms were summed to simulate independent elution (dotted lines). (B) EM monitoring the Cp183 capsid to pass through SRPKΔ-loaded His-trap column. Cp183 appeared as intact capsids either after freely passing through the column (left) or binding to SRPKΔ (right).
(TIF)

Figure S2 **Cryo-EM micrographs of Cp183 Capsid and Cp183 capsid/SRPKΔ.** Cryo-EM images of (a) Cp183 capsid and (b) Cp183 capsid/SRPKΔ embedded in vitreous ice. The Cp183 capsid/SRPKΔ exhibited some thorn-like density (arrows) extending from the particle surface. The scale bar is 50 nm.
(TIF)

Figure S3 **3-D structure of Cp183 capsid/SRPKΔ at lower occupancy.** This sample was obtained from near the midpoint of a Cp183 capsid titration where on average approximately 30 SRPKΔ were bound per Cp183 Capsid. A total of 622 particles were used to compute this 3-D reconstruction to 2.2 nm resolution. Surface-shaded representations of the outer surfaces (a) and inner surfaces (c) of Cp183 capsid/SRPKΔ viewed along the icosahedral twofold axis at 100% expected mass for Cp183 Capsid. The central section of Cp183 capsid/SRPKΔ volume was shown in (b). These results are essentially the same as those shown in figure 3, though at lower resolution. The scale bar is 5 nm.
(TIF)

Figure S4 **Resolution estimated by Fourier shell correlation.** The particles used to compute the final 3-D reconstructions were evenly divided into two sub-datasets and reconstructed. The resolutions of the reconstructions were defined as the spatial frequency where the correlation between Fourier terms was ≤50%.
(TIF)

Text S1 **Derivation of the binding isotherm of SRPK for mixed Cp183 dimer and capsid.**
(DOC)
