# Supplementary figures and images for: A Kinase Chaperones Hepatitis B Virus Capsid Assembly and Captures Capsid Dynamics in vitro
Source: PLoS Pathog. 2011 Nov 17;7(11):e1002388. doi: 10.1371/journal.ppat.1002388 (PMC3219723; doi:10.1371/journal.ppat.1002388)

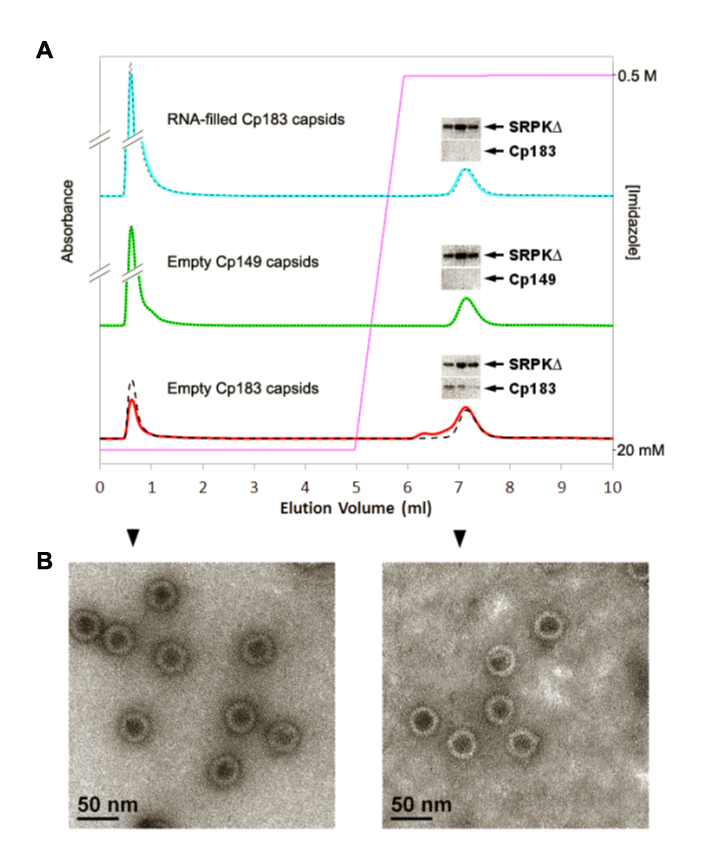

Supplement: Figure S1 — Capsid binding to column-immobilized SRPKΔ. (A) Binding assays to show that empty Cp183 capsids interact with SRPKΔ, in contrast to empty Cp149 capsids and RNA-filled Cp183 capsids. We preloaded His-tagged SRPKΔ onto a His-trap (GE Health Sciences) column, and then ran a capsid solution through. Bound protein was eluted (solid curves) with an imidazole gradient (pink curve); fractions were evaluated by SDS-PAGE (insets). As a control, binding of capsid and SRPKΔ were examined separately, and the chromatograms were summed to simulate independent elution (dotted lines). (B) EM monitoring the Cp183 capsid to pass through SRPKΔ-loaded His-trap column. Cp183 appeared as intact capsids either after freely passing through the column (left) or binding to SRPKΔ (right). (TIF) [file ppat.1002388.s001.tif]

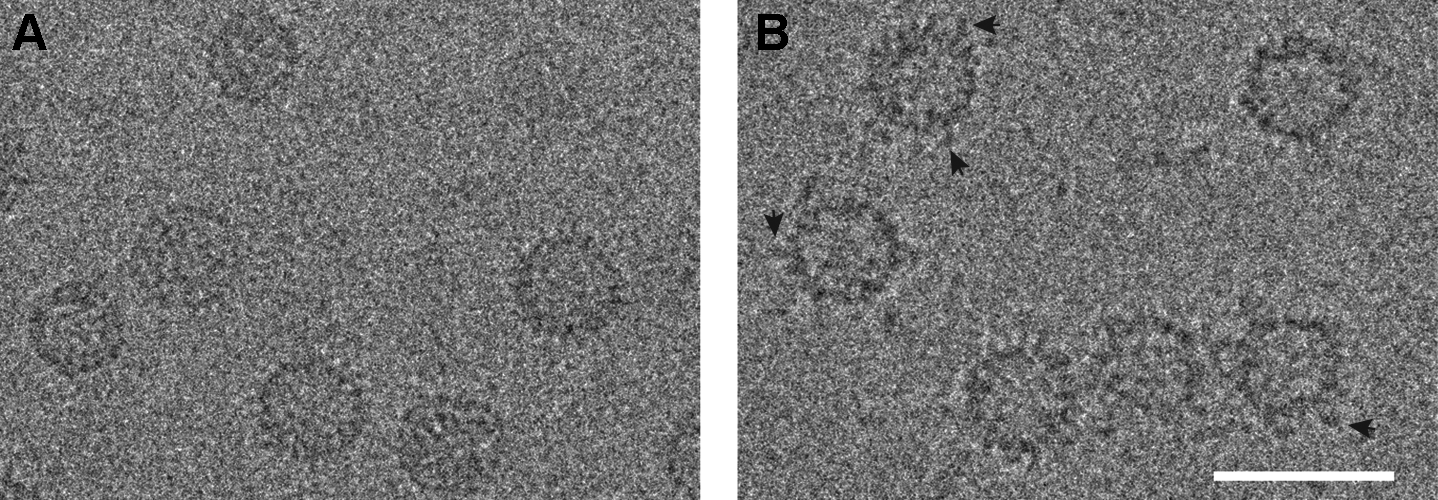

Supplement: Figure S2 — Cryo-EM micrographs of Cp183 Capsid and Cp183 capsid/SRPKΔ. Cryo-EM images of (a) Cp183 capsid and (b) Cp183 capsid/SRPKΔ embedded in vitreous ice. The Cp183 capsid/SRPKΔ exhibited some thorn-like density (arrows) extending from the particle surface. The scale bar is 50 nm. (TIF) [file ppat.1002388.s002.tif]

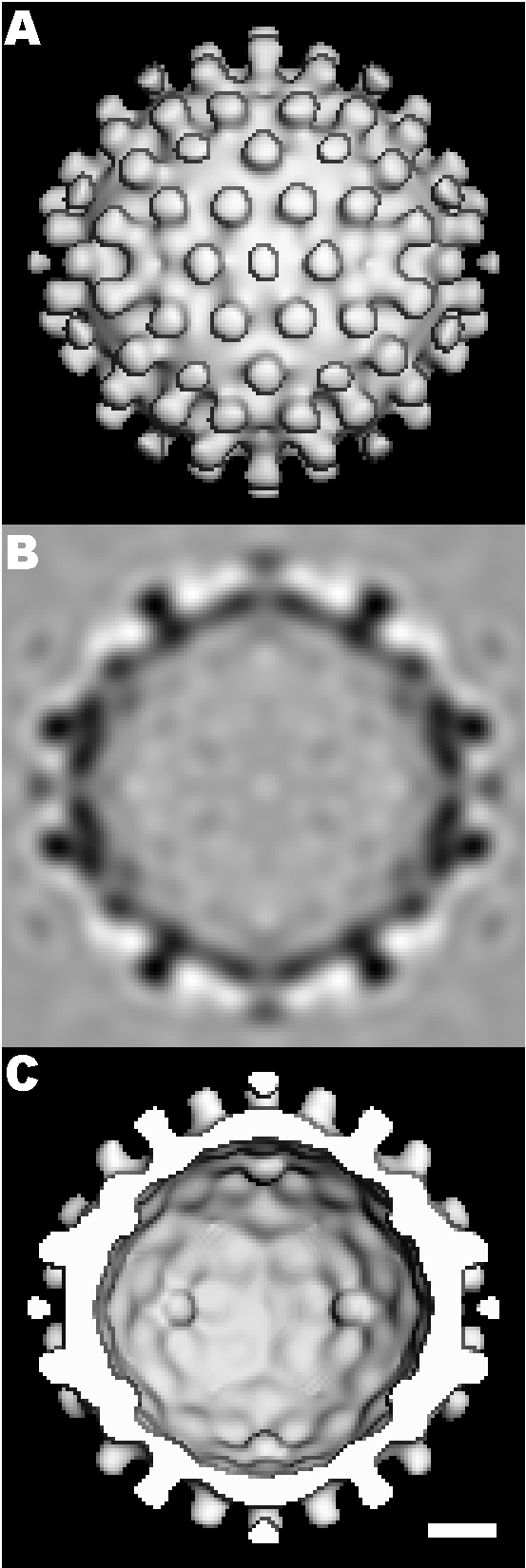

Supplement: Figure S3 — 3-D structure of Cp183 capsid/SRPKΔ at lower occupancy. This sample was obtained from near the midpoint of a Cp183 capsid titration where on average approximately 30 SRPKΔ were bound per Cp183 Capsid. A total of 622 particles were used to compute this 3-D reconstruction to 2.2 nm resolution. Surface-shaded representations of the outer surfaces (a) and inner surfaces (c) of Cp183 capsid/SRPKΔ viewed along the icosahedral twofold axis at 100% expected mass for Cp183 Capsid. The central section of Cp183 capsid/SRPKΔ volume was shown in (b). These results are essentially the same as those shown in figure 3, though at lower resolution. The scale bar is 5 nm. (TIF) [file ppat.1002388.s003.tif]

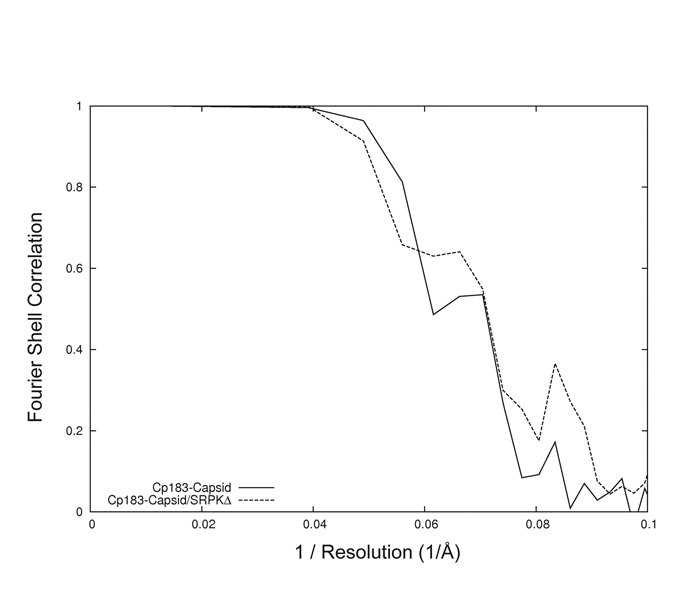

Supplement: Figure S4 — Resolution estimated by Fourier shell correlation. The particles used to compute the final 3-D reconstructions were evenly divided into two sub-datasets and reconstructed. The resolutions of the reconstructions were defined as the spatial frequency where the correlation between Fourier terms was ≤50%. (TIF) [file ppat.1002388.s004.tif]
