# Supplementary material for: A Kinase Chaperones Hepatitis B Virus Capsid Assembly and Captures Capsid Dynamics in vitro
Source: PLoS Pathog. 2011 Nov 17;7(11):e1002388. doi: 10.1371/journal.ppat.1002388 (PMC3219723; doi:10.1371/journal.ppat.1002388)
Supplement: Text S1 — Derivation of the binding isotherm of SRPK for mixed Cp183 dimer and capsid. (DOC) [file ppat.1002388.s005.doc]

**Derivation of the SRPK∆ binding curve**

The binding curve shown in figure 2 was based sedimenting SRPK∆-capsid complexes and determining the non-sedimenting SRPK∆ remaining in solution. This experiment was used to determine the number of SRPK∆ binding sites per capsid, *N*, and their dissociation constant, *K_D_*. The number of SRPK∆ sediment per capsid, *n*, is thus a function of free SRPK∆, [S].

S1)

However, the concentration of non-sedimenting SRPK, [S]_A_, included both free SRPK∆ and SRPK∆ bound to CP183 dimer.

S2) $\left[S\right]+\left[S\right]_{dimer}=\left[S\right]_{A}$

Cp183 dimer has a concentration of *C’* and each dimer binds up to two SRPK∆ molecules with a dissociation constant of *K’_D_*. The appropriate binding expression can be substituted into equation S2 and rearranged to a polynomial.

S3) $\begin{matrix} \left[S\right]+2\frac{\left[S\right]}{\left[S\right]+K_{D}}C'=\left[S\right]_{A} \\ \\ \left[S\right]^{2}+\left(2C'+K'_{D}-\left[S\right]_{A}\right)\left[S\right]-K'_{D}\left[S\right]=0 \end{matrix}$

The concentration of free SRPK∆, from the quadratic equation, can be substituted into equation S1 to yield equation 5 in the text.

S4) $\begin{matrix} \left[S\right]=\frac{-\left(2C'+K'_{D}-\left[S\right]_{A}\right)+\sqrt{\left(2C'+K'_{D}-\left[S\right]_{A}\right)^{2}+4K'_{D}\left[S\right]_{A}}}{2} \\ n=\frac{N}{\frac{K_{D}}{\left[S\right]}+1}=\frac{N}{\frac{K_{D}}{\frac{-\left(2C'+K'_{D}-\left[S\right]_{A}\right)+\sqrt{\left(2C'+K'_{D}-\left[S\right]_{A}\right)^{2}+4K'_{D}\left[S\right]_{A}}}{2}}+1} \\ n=\frac{N}{\frac{2K_{D}}{-\left(2C'+K'_{D}-\left[S\right]_{A}\right)+\sqrt{\left(2C'+K'_{D}-\left[S\right]_{A}\right)^{2}+4K'_{D}\left[S\right]_{A}}}+1} \end{matrix}$
